# Clinical Characteristics, Management, and Outcomes in Cardiogenic Shock: Insights From a High-Volume Italian Cardiac Intensive Care Unit

**DOI:** 10.1097/FJC.0000000000001584

**Published:** 2024-08-06

**Authors:** Marco Giuseppe Del Buono, Giulia La Vecchia, Alessia D'Aiello, Daniela Pedicino, Gaetano Pinnacchio, Lorenzo Genuardi, Rocco Antonio Montone, Gianluigi Saponara, Antonio Di Renzo, Cristina Conte, Francesco Cribari, Francesco Moroni, Simone Filomia, Mattia Brecciaroli, Cristina Aurigemma, Giovanna Liuzzo, Carlo Trani, Francesco Burzotta, Tommaso Sanna

**Affiliations:** * Department of Cardiovascular and Pulmonary Sciences, Catholic University of the Sacred Heart, Rome, Italy;; † Department of Cardiovascular Medicine, Fondazione Policlinico Universitario A. Gemelli IRCCS, Rome, Italy;; ‡ Operative Unit of Diagnostic Interventional Cardiology, Isola Tiberina-Gemelli Isola, Rome, Italy; and; § Robert M. Berne Cardiovascular Research Center, Department of Medicine, Division of Cardiovascular Medicine, Heart and Vascular Center, University of Virginia, Charlottesville, VA.

**Keywords:** cardiogenic shock, Italy, heart failure, mortality, SCAI, epidemiology

## Abstract

Cardiogenic shock (CS) is a life-threatening condition. The aim of this study is to evaluate the clinical characteristics, management, and complication rate of patients with CS admitted to a high-volume hospital in Italy. We retrospectively reviewed the clinical, echocardiographic, and laboratory data, therapeutic management, and outcomes of patients with CS admitted to the Policlinico Gemelli (Rome) between January 1, 2020, and January 1, 2023. We included 96 patients [median age 71 years, interquartile range 60–79; 65 (68%) males], of whom 49 patients (51%) presented CS secondary to acute myocardial infarction and 60 (63%) with a de novo presentation of CS. Dobutamine was the most frequently used inotrope and noradrenaline the most frequently used vasopressor (adopted in 56% and 82% of cases, respectively). Forty-five (47%) patients died during the hospitalization. Nonsurvivors were older and had a higher inflammatory burden at admission, elevated lactate levels, a greater increase in lactate levels, higher left ventricular filling pressures, and worse right ventricular function. C-reactive protein levels [odds ratio (OR) 1.03, 95% confidence interval (CI) (1.00–1.04), *P* = 0.027], lactate levels at admission (OR 3.49, 95% CI, 1.59–7.63, *P* = 0.02), and increase in lactate levels (OR 2.8, 95% CI, 1.37–5.75, *P* = 0.005) were independent predictors of in-hospital all-cause death. Our data contribute to the assessment of the regional variations in the management and outcomes of patients with CS. We observed a high mortality and complication rate. Lactate acidosis and C-reactive protein measured at admission may help in identifying patients at higher risk of adverse in-hospital outcomes.

## INTRODUCTION

Cardiogenic shock (CS) is a life-threatening medical condition that occurs when, as a consequence of cardiac disease, the physiological interdependence between oxygen delivery and consumption is lost, leading to multiorgan failure.^1^ Despite significant advances in the management of cardiovascular diseases, CS remains a major cause of morbidity and mortality worldwide. Interestingly, variability in etiology, treatment, and outcomes have been reported, translating into an in-hospital mortality rate of 30%–50% in contemporary series,^2–6^ but this ranged from 2.2% to 62.5% as patients varied from less severe to more advanced shock stages.^7^ Several studies have been published, contributing to a better characterization of this clinical heterogeneity. However, the epidemiology of CS in Italy is still poorly described because of the lack of a robust and structured CS network, shared healthcare protocols, and electronic health record systems.^8–10^ The Altshock‐2 Registry, a multicenter prospective data collection with 11 Italian Centers contributing to patients' enrollment, started recruiting patients in March 2020 and is, to date, the only registry of patients with CS in Italy.^11,12^

The aim of this study is to analyze the clinical characteristics at admission, the clinical course, the treatments received, the outcomes observed, and their predictors in a series of consecutive patients with a diagnosis of CS admitted to an academic, high-volume, tertiary referral cardiac intensive care unit (CICU) in Italy.

## METHODS

The Fondazione Policlinico Universitario Agostino Gemelli IRCCS (Rome, Italy) is a surgical/interventional tertiary center without an active heart transplant and left ventricular assist device (LVAD) program. The hospital has a CICU, a general intensive care unit, a postcardiac surgery intensive care unit, a neurological intensive care unit and a postoperative general ICU.

Electronic health records were retrospectively reviewed to identify cases of CS admitted to the CICU of the Fondazione Policlinico Universitario Agostino Gemelli IRCCS between January 1, 2020, and January 1, 2023.

The initial search was based on the International Statistical Classification of Diseases and Related Health Problems (ICD-9) code for CS (785.51) collected in our electronic health record system (Digistat, GE Healthcare). Two cardiologists performed a review to confirm the diagnosis of CS, and data abstraction from electronic health records for a database including demographic data, clinical characteristics, laboratory data, diagnostic test results, treatments received, and in-hospital outcomes and complications. Disagreements on findings or readings were resolved through discussion and consensus. For this analysis, we included patients with CS stage C or worse according to the Society for Cardiovascular Angiography and Interventions (SCAI) shock classification.^13^ In brief, SCAI SHOCK stage A includes stable patients with cardiac problems at risk for CS but failing to meet the preshock (stage B) or shock (stages C–E) criteria. Stage B represents patients who have evidence of hemodynamic instability (hypotension or compensatory tachycardia) without hypoperfusion (lactate level of <2 mmol/L); stage C represents the classic patients with CS who present with hypoperfusion (lactate level of >2 mmol/L) either untreated or requiring hemodynamic support through pharmacologic or mechanical intervention; stage D represents the failure of initial therapy and the need to add 1 or more additional vasoactive drugs or mechanical circulatory support devices; stage E is reserved for refractory shock with actual or impending cardiovascular collapse despite high and escalating levels of support (including arrest in progress). The CardShock risk score was calculated at admission and consists of 7 variables giving a maximum of 9 points [including age >75 years, confusion at presentation, history of previous acute myocardial infarction (AMI) or coronary bypass graft surgery, AMI-related CS, left ventricular ejection fraction (LVEF) <40%, blood lactate, kidney function].

Patients with alternative causes of shock (ie, hypovolemic, septic, hemorrhagic, obstructive), on antibiotic therapy before or at the time of the admission for suspected sepsis, and those who were candidates to palliative care because of other concomitant severe health conditions or patient's advanced directive were excluded from this study.

The aim of this study is to assess how the clinical characteristics at admission and the treatments received may affect the clinical course and the outcomes observed.

The primary outcome of interest was all-cause mortality. Secondary outcomes included resuscitated in-hospital cardiac arrest (defined as cardiac arrest occurring during the hospital stay with prompt resuscitation with chest compressions, defibrillation, or both), acute cerebrovascular accident (defined clinically and/or using brain imaging), deep vein thrombosis or pulmonary embolism (as documented using Doppler venous ultrasound or chest computed tomography scan), need for renal replacement therapy (ie, continuous renal replacement therapy for acute or worsening renal failure), major bleedings [defined according to the International Society on Thrombosis and Haemostasis as fatal bleeding, and/or symptomatic bleeding in a critical area or organ, such as intracranial, intraspinal, intraocular, retroperitoneal, intra-articular or pericardial, or intramuscular with compartment syndrome, and/or bleeding causing a fall in hemoglobin levels of 1.24 mmol/L (2 g/dL or greater) or more, or leading to a transfusion of 2 U or more of whole blood or red cells], delirium (defined as a disturbance of consciousness and cognition that develops over a short period of time and fluctuating over time with need of nonpharmacological and pharmacological interventions), need for endotracheal intubation, and infection (suspected or confirmed and needing antibiotic therapy).

### Statistical Analysis

Descriptive statistics were used to describe the characteristics of the study participants. Continuous data were reported as median and interquartile range, and data were compared with the Mann–Whitney *U* test or Kruskal–Wallis test, as appropriate. Categorical variables were expressed as numbers and percentages (%) and compared using χ^2^ test or Fisher exact test, as appropriate. Correlations between clinical variables were determined by Spearman's correlation coefficients. A logistic regression analysis for in-hospital mortality was performed including clinically significant variables. Those meeting statistical significance in the univariable model (*P* < 0.05, 2-tailed) were further evaluated in the multivariable logistic regression model to identify independent variables associated with the occurrence of in-hospital mortality. For the univariable and multivariable analysis, the measure of association was expressed as an odds ratio (OR) and 95% confidence interval (CI). Receiver-operating characteristic (ROC) curve analysis was used to estimate the overall predictive accuracy of continuous variables of interest to define the optimal predictive cut-off value for in-hospital all-cause mortality by evaluating the area under the curve (AUC) and the respective 95% CI. All the analyses were completed using SPSS, version 29.0 (SPSS, Chicago, IL).

This study complies with the Declaration of Helsinki and was approved by the Catholic University Ethics Committee (protocol number ID 5285).

## RESULTS

### Study Population

A total of 141 consecutive patients were identified using the ICD-9 for CS (785.51). After reviewing electronic health records, 45 patients were considered ineligible for our study. Fourteen patients did not qualify for inclusion (SCAI stage lower than C during the whole hospital stay), and 31 presented at least 1 exclusion criterion (18 septic shock, 8 obstructive shock, and 5 patients opted for comfort measures).^13^ A final cohort of 96 patients with a confirmed diagnosis of CS SCAI stage C or worse was analyzed for this study.

### Baseline Characteristics

**FIGURE 1.**
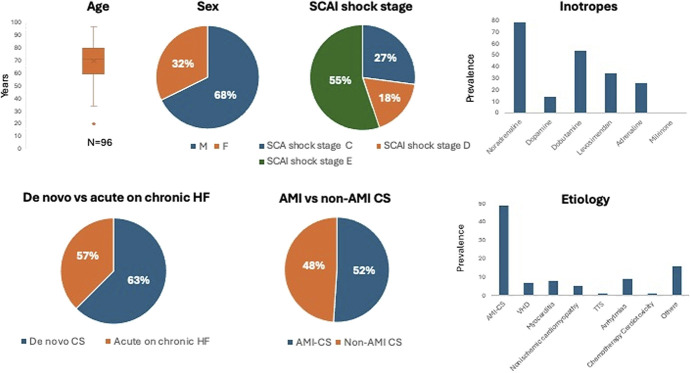
Clinical characteristics of the population cohort of patients with CS. This figure illustrates the demographic characteristics (age, sex, comorbidities), the clinical presentations on admission according to the SCAI shock stage, the etiology, and the treatments administrated in our cohort of patients with CS.

Baseline demographic, clinical, laboratory, and echocardiographic data and treatments received are summarized in Table 1 and Figure 1. The median age was 71 years (interquartile range 60–79); 65 (68%) were men, and 91 (95%) were White. Clinical presentation was de novo *CS* in 60 patients and *acute decompensation of chronic heart failure* (ADHF) in 36 (63% vs. 37%, respectively). Etiology of CS was AMI in 49 patients (51%), valvular heart disease (VHD) in 7 (7%), nonischemic cardiomyopathy in 5 (5%), myocarditis in 8 (8%), Takotsubo syndrome in 1 (1%), life-threatening arrhythmias in 9 (9%), adverse reaction to chemotherapy in 1 (1%), and other causes in 16 patients (17%).

**TABLE 1.** Baseline Characteristics of Patients Admitted With CS

Variable	Total (n = 96)
Age (y)	71 [60–79]
Sex (F), n (%)	31 (32)
White	91 (95)
Black	2 (2)
Other	3 (3)
BMI (kg/m^2^)	24 [22–27]
Diabetes, n (%)	19 (20)
COPD n (%)	19 (20)
CKD, n (%)	17 (18)
Active cancer n (%)	6 (6)
CVA, n (%)	7 (7)
Clinical presentation	
SCAI stage at admission, n (%)	
A	3 (3)
B	30 (31)
C	39 (41)
D	16 (∼17)
E	8 (8)
Worst SCAI stage during the hospital stay, n (%)	
C	26 (27)
D	17 (18)
E	53 (55)
CardShock risk score	4 (3–5)
De novo cardiogenic shock, n (%)	60 (63)
Acute on chronic heart failure, n (%)	36 (37)
Etiologies, n (%)	
AMI-CS	49 (51)
VHD	7 (7)
Myocarditis	8 (8)
Nonischemic cardiomyopathy	5 (5)
TTS	1 (1)
Arrhytmias	9 (9)
Chemotherapy cardiotoxicity	1 (1)
Other	16 (17)
Laboratory data	
WBC 10^9^/L	11.6 [7.7–15.5]
CRP mg/L	31.0 [8.9–111.0]
PCT ng/mL	3.0 [1.0–17.2]
Lactic acid mmol/L	4.0 [1.9–7.0]
TnI (peak) ng/L	28.5 [10.8–52.3]
NTproBNP pg/mL	21,500 [1000–45,250]
Echocardiography at admission	
LVEF (%)	25 [20–35]
EDV (mL)	86 [65–101]
LVOT VTI (cm)	11 [9–12]
E/e′	15 [13–22]
TAPSE (mm)	14 [11–18]
sPAP (mm Hg)	45 [35–55]
In-hospital treatment	
Necessity of inotropes/vasopressors, n (%)	100 (100)
Noradrenaline	79 (82)
Dopamine	14 (15)
Dobutamine	54 (56)
Levosimendan	35 (37)
Adrenaline	26 (27)
Milrinone	1 (1)
Necessity of temporary ventricular support device, n (%)	34 (35)
IABP	18 (19)
Impella	22 (23)
ECMO	6 (6)
Necessity of vasodilators, n (%)	16 (17)
Necessity of diuretics, n (%)	82 (85)
Treatment with high-dose corticosteroids, n (%)	8 (8)
Pressure-support ventilation	41 (43)
HFNC	10 (10)
Both PSV and HFNC	4 (4)

AS, aortic stenosis; BMI, body mass index; CKD, chronic kidney disease; CVA, cerebrovascular accident; ECMO, extracorporeal membrane oxygenation; EDV, end-diastolic volume; ESV, end-systolic volume; HFNC, high-flow nasal cannula; IABP, intra-aortic balloon pump; LVOT, left ventricular outflow tract velocity–time integral; NT proBNP, *N*-terminal pro-B-type natriuretic peptide; PCT, procalcitonin; sPAP, systolic pulmonary artery pressure; TTS, Takostubo syndrome; WBC, white blood cell count.

SCAI stage at admission was A in 3 (3%), B in 30 (31%), C in 39 (41%), D in 16 (∼17%), and E in 8 patients (8%). The median Card Shock risk score was 4 [3–5]. The Sequential Organ Failure Assessment score within 24 hours was 8 [6–9].

The median LVEF was 25 [20–35] % with a median left ventricular outflow tract velocity–time integral of 11 [9–12] cm and a median E/e' average ratio of 15 [13–22].

Lactate levels at admission were 4 [1.9–7] mmol/L, median C-reactive protein (CRP) at admission was 31.0 [8.9–111.0] mg/L, and white blood cell counts were 11.6 [7.7–15.5] 10^9^/L.

### Clinical Course and Treatment Received

The hospital length of stay was 11.5^2–20^ days. During the hospital stay, 26 patients (27%) progressed to SCAI stage C, 17 (18%) to stage D, and 53 (55%) to SCAI stage E. All patients required inotropes and/or vasopressors as detailed in Table 1. In particular, dobutamine and levosimendan were the most frequently used inotropes [adopted in 54 (56%) patients and 35 (37%) patients, respectively] and noradrenaline the most frequently used vasopressor [adopted in 79 (82%) patients]. Most patients received diuretics [82 (85%)], while only 16 (17%) patients required vasodilators and 8 (8%) patients high-dose corticosteroids. Mechanical circulatory support was required in 34 patients (35%), including 18 (19%) intra-aortic balloon pump, 22 (23%) percutaneous LVAD [p-LVAD (Impella CP; Abiomed)], and 6 (6%) venous-arterial extracorporeal membrane oxygenation. A pulmonary artery catheter was used in 15 (16%) of patients for invasive hemodynamic monitoring.

### Study Outcomes

All-cause in-hospital mortality occurred in 45 (47%) patients, of whom 1 (2%) patient had presented with SCAI stage A, 10 (22%) patients with SCAI stage B, 18 (40%) with SCAI stage C, 12 (27%) with SCAI stage D, and 4 (9%) with SCAI stage E at admission.

**FIGURE 2.**
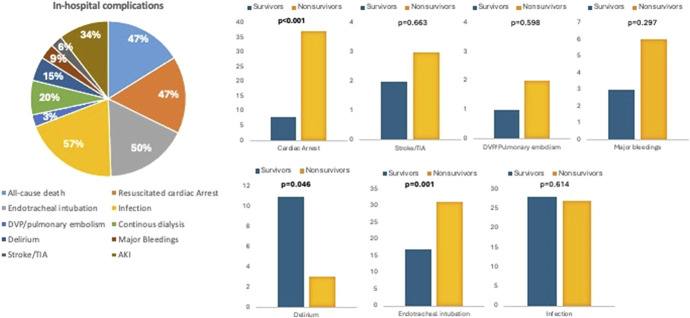
Prevalence of in-hospital complications among patients with CS. This figure illustrates the distribution of in-hospital complications among our population cohort of patients with CS and survivors versus nonsurvivors.

Regarding the secondary outcomes of interest, cardiac arrest occurred in 45 patients (47%), infection in 55 (57%) patients, major bleedings in 9 (9%) patients, delirium occurred in 14 (15%) patients, and deep venous thrombosis or pulmonary embolism in 3 (3%) patients. Furthermore, 20 (20%) patients needed renal replacement therapy and 48 (50%) patients needed endotracheal intubation (Fig. 2).

The characteristics of survivors (Ss) versus nonsurvivors (NSs) are summarized in Table 2. In brief, NSs were older [median age 75 (63–82) years vs. 69 (56–75) years; *P* = 0.019], had a higher prevalence of chronic obstructive pulmonary disease (COPD) [13 (29%) vs. 6 (12%), *P* = 0.036], presented higher levels of white blood cell at admission [13.4 (8.1–18.9) 10^9^/L vs. 9.5 (7.3–14.2) 10^9^/L, *P* = 0.019], CRP [60 (27.0–128.5) mg/L vs. 7.7 (2.8–31.0) mg/L, *P* < 0.001], procalcitonin [1.8 (0.4–5.5) ng/mL vs. 0.2 (0.05–0.31) ng/mL, *P* < 0.001], *N*-terminal pro-BNP type natriuretic peptide (NT-pro BNP) [13,037 (8129–31,935) pg/mL vs. 9220 (4311–20,199) pg/mL, *P* = 0.043], and lactate [6 (3.0–9.8) mmol/L vs. 3 (1.8–4.8) mmol/L, *P* = 0.001] at admission, and showed a higher increase in lactate levels at 24–48 hours from admission (delta with baseline lactate levels) [+2.25 (−1.5; 5) vs. −1.05 (−2.8; 0.0), *P* = 0.001]. NSs had a worse SCAI stage at hospital admission (*P* < 0.001), a more depressed right ventricular (RV) function, assessed by tricuspid annular plane systolic excursion (TAPSE) [12 (10–15) mm vs. 18 (12–19) mm, *P* < 0.001], and a higher estimated left ventricular filling pressure assessed by the average E/e′ ratio [21 (18–25) vs. 14 (11–18), *P* < 0.001]. NSs had a higher CardShock risk score [5 (3.5–6) vs. 3 (2.8–4), *P* < 0.001].^14^

**TABLE 2.** Baseline Characteristics in Survivors Versus Nonsurvivor Patients With CS

	Survivors (n = 51)	Nonsurvivors (n = 45)	*P*
Age (y)	69 [56–75]	75 [63–82]	0.019
Sex (F), n (%)	14 (27)	17 (38)	0.280
BMI (kg/m^2^)	25 [23–27]	24 [21–26]	0.316
Diabetes, n (%)	10 (20)	9 (20)	0.962
COPD, n (%)	6 (12)	13 (29)	0.036
CKD, n (%)	9 (18)	8 (18)	0.987
Active cancer—n (%)	3 (6)	3 (7)	1.000
CVA—n (%)	2 (4)	5 (11)	0.247
Clinical presentation			
SCAI stage—at admission, n (%)			0.083
A	2 (4)	1 (2)	
B	20 (39)	10 (22)	
C	21 (42)	18 (40)	
D	4 (8)	12 (27)	
E	4 (8)	4 (9)	
Worse SCAI stage, n (%)			<0.001
C	26 (51)	0 (0)	
D	17 (33)	0 (0)	
E	8 (16)	45 (100)	
CardShock risk score	3 (2.8–4)	5 (3.5–6)	<0.001
No previous history of heart disease, n (%)	15 (29)	21 (47)	0.081
Etiologies, n (%)			0.513
AMI-CS	24 (47)	23 (51)	
VHD	3 (6)	2 (4)	
Myocarditis	6 (12)	2 (4)	
Nonischemic cardiomyopathy	3 (6)	2 (4)	0.817
TTS	0 (0)	1 (2)	0.888
Arrhythmias	6 (12)	5 (11)	0.504
Chemotherapy cardiotoxicity	1 (2)	0 (0)	0.888
Other	8 (16)	8 (18)	0.660
Laboratory test			
WBC 10^9^/L	9.5 [7.3–14.2]	13.4 [8.1–18.9]	0.019
CRP mg/L	7.7 [2.8–31.0]	60.0 [27.0–128.5]	<0.001
PCT ng/mL	0.2 [0.05–0.31]	1.80 [0.4–5.5]	<0.001
Lactic acid mmol/L	3.0 [1.8–4.8]	6.00 [3.0–9.8]	0.001
TnI (peak) ng/L	−1.05 [−2.8 to 0.0]	+2.25 [−1.5 to 5.0]	<0.001
NTproBNP pg/mL	3719 [254.5–40,109.0]	6648 [224.7–36,801.5]	0.848
WBC 10^9^/L	9220 [4311–20,199]	13,037 [8129–31,935]	0.043
Echocardiography at admission			
LVEF (%)	25 [20–35]	25 [19–39]	0.734
EDV (mL)	80 [62–103]	87 [58–100]	0.713
LVOT VTI (cm)	11 [9–15]	10 [9–11]	0.076
E/e′*	14 [11–18]	21 [18–25]	<0.001
TAPSE (mm)	18 [12–19]	12 [10–15]	<0.001
sPAP (mm Hg)	45 [30–54]	50 [45–60]	0.055
In-hospital treatment			
Necessity of temporary ventricular support device, n (%)	18 (35)	16 (36)	0.979
Necessity of vasodilators, n (%)	11 (22)	5 (11)	0.170
Treatment with high-dose corticosteroids, n (%)	5 (10)	3 (7)	0.197

* E/e′ was missing in 13 patients.

AS, aortic stenosis; BMI, body mass index; CKD, chronic kidney disease; CVA, cerebrovascular accident; EDV, end-diastolic volume; ESV, end-systolic volume; HFNC, high-flow nasal cannula; IABP, intra-aortic balloon pump; LVOT, left ventricular outflow tract velocity–time integral; NT proBNP, N-terminal pro-B-type natriuretic peptide; PCT, procalcitonin; sPAP, systolic pulmonary artery pressure; TTS, Takostubo syndrome; WBC, white blood cell count.

Moreover, they presented more often a resuscitated in-hospital cardiac arrest [37 (82%) vs. 8 (16%), *P* < 0.001], the necessity of endotracheal intubation [31 (69%) vs. 17 (33%), *P* = 0.001], and a lower rate of delirium compared with Ss [3 (7%) vs. 11 (22%), *P* = 0.046]. On the contrary, no differences were found among Ss versus NSs in the etiologies of CS (*P* > 0.05), in LVEF values (*P* = 0.734), in the inotropes/vasopressors used, or in the necessity of temporary ventricular support [Ss 18 (33%) vs. NSs 16 (36%), *P* = 0.979], as well as in transient ischemic attack/stroke, deep venous thrombosis or pulmonary embolism, kidney failure requiring renal replacement therapy, major bleedings, and infection rate. The results are summarized in Table 3.

**TABLE 3.** In-Hospital Complications in Survivors and Nonsurvivor Patients

	Survivors (n = 51)	Nonsurvivors (n = 45)	*P*
Resuscitated IHCA, n (%)	8 (16)	37 (82)	<0.001
Stroke or TIA, n (%)	2 (4)	3 (7)	0.663
DVT or PE, n (%)	1 (2)	2 (2)	0.598
Endotracheal intubation, n (%)	17 (33)	31 (69)	0.001
Major bleedings, n (%)	3 (6)	6 (13)	0.297
Delirium, n (%)	11 (22)	3 (7)	0.046
Infection needing antibiotics, n (%)	28 (55)	27 (60)	0.614
Continuous dialysis, n (%)	9 (18)	11 (24)	0.413
Acute kidney injury, n (%)	11 (22)	18 (40)	0.041

DVT, deep vein thrombosis; IHCA, in-hospital cardiac arrest; PE, pulmonary embolism; TIA, transient ischemic attack.

### Predictors of In-Hospital Mortality

At univariate logistic regression, age, lactate levels at admission, lactate increase in the first 48 hours, CRP levels at admission, and TAPSE were predictors of in-hospital death for all causes (Table 4).

**TABLE 4.** Variables Associated With In-Hospital Mortality by Univariate and Multivariate Logistic Regression Analysis

Variables	Univariable Analysis		Multivariable Analysis	
	OR (95% CI)	*P*	OR (95% CI)	*P*
Age (y)	1.04 [1.01–1.08]	0.010	1.09 [0.99–1.22]	0.091
Lactate levels (mmol/L)	1.23 [1.08–1.39]	0.002	3.49 [1.59–7.63]	0.020
Delta lactate	1.17 [1.04–1.32]	0.009	2.8 [1.37–5.75]	0.005
NTproBNP (pg/mL)	1.00 [1.00–1.00]	0.161		
CRP (mg/L)	1.01 [1.01–1.02]	0.001	1.03 [1.00–1.04]	0.027
WBC (10^9^/L)	1.07 [0.99–1.16]	0.063		
PCT (ng/mL)	1.13 [0.93–1.37]	0.212		
TAPSE (mm)	0.81 [0.72–0.92]	0.001	0.79 [0.59–1.04]	0.091

NT proBNP, *N*-terminal pro-B-type natriuretic peptide; PCT, procalcitonin; WBC, white blood cell count.

At multivariate analysis, lactate levels at admission [OR 3.49 per unit in increase mmol/L (1.59–7.63), *P* = 0.02], lactate increase in the first 48 hours [OR 2.8 per unit in increase mmol/L (1.37–5.75), *P* = 0.005], and CRP levels at admission [OR 1.03 per unit in increase mg/L (1.00–1.04), *P* = 0.027] remained independent predictors of in-hospital death for all causes. The E/e′ ratio was not included in the analysis because it was missing in 13 patients (Table 4).

**FIGURE 3.**
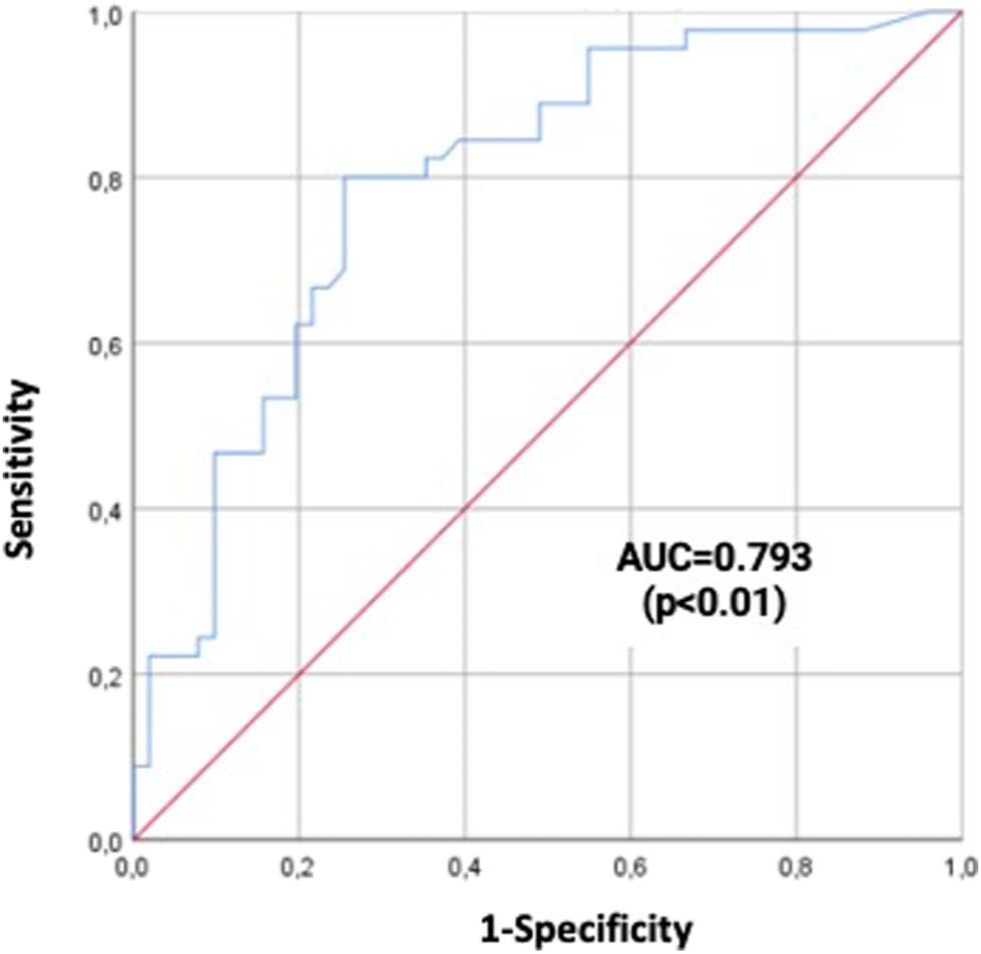
ROC curve analysis for CRP to predict in-hospital complications. ROC curve analysis showed an AUC for CRP levels to predict in-hospital mortality of 0.793 (*P* < 0.01).

**FIGURE 4.**
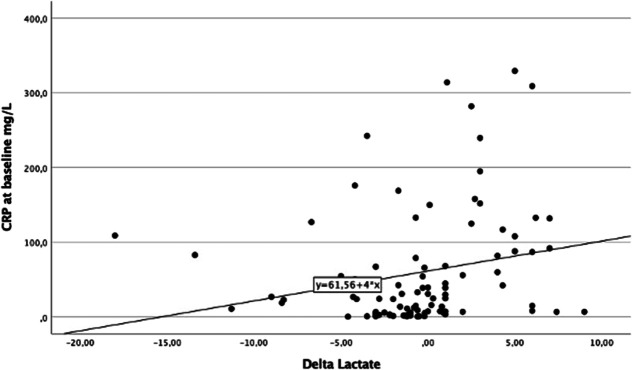
Correlation between CRP at admission and delta interval changes in lactic acid levels. The scatterplot graph illustrates the correlation between delta interval changes in lactic acid levels and CRP at admission (R = +0.326, *P* = 0.01).

ROC curve analysis was performed to assess the ability of CRP at admission to predict in-hospital mortality. The AUC for CRP levels to predict in-hospital mortality was 0.793 (*P* < 0.01; Fig. 3). The optimal cut-off was identified using the Youden method and was found to be a CRP cut-off value of 25 mg/L (sensitivity of 82% and specificity of 64%). Of note, 50 (52%) of the patients had CRP on presentation ≥25 mg/L. In-hospital mortality occurred in 9 (20%) patients with CRP levels <25 mg/L and 36 (72%) patients among those with CRP levels equal or above the cut-off value (*P* < 0.001). Table 5 summarizes the clinical, laboratory, and echocardiographic data of the overall population divided for the cut-off of CRP of ≥25 mg/L. We did not find a significant correlation between lactic acid level at admission and CRP levels (R = +0.175, *P* = 0.101); however, we found a moderate correlation between delta interval changes in lactic acid levels and CRP at admission (R = +0.326, *P* = 0.01), with higher levels of CRP at admission correlating with higher lactate increases (Fig. 4).

**TABLE 5.** Baseline Characteristics and Treatments Received According to the Cut-Off Value of CRP

	CRP <25 mg/L (n = 46)	CRP ≥25 mg/L (n = 50)	*P*
Age (y)	68 [58–75]	71 [58–79]	0.596
Sex (F), n (%)	15 (33)	16 (32)	0.949
BMI (kg/m^2^)	24 [23–28]	23 [21–26]	0.381
Diabetes, n (%)	12 (26)	7 (14)	0.138
COPD, n (%)	11 (24)	8 (16)	0.331
CKD, n (%)	11 (24)	6 (12)	0.127
Active cancer n (%)	2 (4)	4 (8)	0.679
CVA, n (%)	3 (6)	4 (8)	1.000
Clinical presentation			
Worse SCAI stage, n (%)			<0.001
C	22 (48)	4 (8)	
D	10 (22)	7 (14)	
E	14 (30)	39 (78)	
Card shock	4 (3–4)	5 (3–5.2)	0.07
No previous history of heart disease, n (%)	19 (41)	28 (61)	0.460
Etiologies, n (%)			0.553
AMI-CS	22 (48)	27 (54)	
VHD	4 (9)	3 (6)	
Myocarditis	3 (6)	5 (10)	
TTS	0	1 (2)	
Other	17 (37)	14 (28)	
Laboratory test			
WBC, 10^9^/L	8 [6.6–13]	13.4 [9.2–17.7]	0.004
CRP, mg/L*	7.6 [3.8–13.8]	87 [42.4–156.5]	<0.001
PCT, ng/mL	2.0 [1.0–8.7]	13.0 [3.0–26.7]	0.001
Lactic acid, mmol/L	3.0 [1.7–4.8]	4.1 [2.0–7.4]	0.042
TnI (peak), ng/L	26.5 [8.5–45.7]	26.5 [12.0–45.7]	0.774
NTproBNP, pg/mL	17.5 [5.5–40.0]	25.0 [1.5–41.5]	0.938
Echocardiography at admission			
LVEF (%)	25 [20–35]	23 [16–40]	0.980
EDV (mL)	87 [55–100]	92 [70–109]	0.475
ESV (mL)	33 [42–42]	99 [80–107]	0.480
LVOT VTI (cm)	10 [8–11]	11 [9–13]	0.491
E/e′	16 [14–22]	18 [12–22]	0.706
TAPSE (mm)	15 [11–18]	12 [10–17]	0.315
sPAP (mm Hg)	45 [32–50]	50 [45–59]	0.053
In-hospital treatment			
Necessity of inotropes, n (%)	46 (100)	50 (100)	—
Necessity of temporary ventricular support device, n (%)	12 (26)	22 (44)	0.067
Necessity of vasodilators, n (%)	10 (22)	6 (12)	0.020
Necessity of diuretics, n (%)	38 (83)	44 (88)	0.455
Treatment with high-dose corticosteroids, n (%)	6 (13)	5 (10)	0.640
Necessity of NIV, n (%)			0.561
NIV	21 (46)	20 (40)	
HFNC	5 (11)	5 (10)	
Both NIV and HFNC	3 (6)	1 (2)	

*Please note that CRP value is available only in 80 of 96 patients.

AS, aortic stenosis; BMI, body mass index; CKD, chronic kidney disease; CVA, cerebrovascular accident; EDV, end-diastolic volume; ESV, end-systolic volume; HFNC, high-flow nasal cannula; IABP, intra-aortic balloon pump; LVOT, left ventricular outflow tract velocity–time integral; NT proBNP, *N*-terminal pro-B-type natriuretic peptide; sPAP, systolic pulmonary artery pressure; TTS, Takostubo syndrome; WBC, white blood cell count.

## DISCUSSION

Our research contributes to the assessment of regional differences observed in published reports of CS. We conducted a thorough analysis of a group of patients with CS in an Italian CICU to collect data on clinical characteristics upon admission, clinical course, treatments received, observed outcomes, and predictors of prognosis.

The most frequent presentation of CS was de novo CS, with AMI being the most common cause, followed by VHD and myocarditis. This finding is in line with published evidence and suggests the opportunity to prioritize the development of locally standardized approaches to these specific presentations of CS.^1,4,5,12–14^ All patients were given inotropes and vasopressors, with dobutamine and noradrenaline being the most used drugs. Despite the availability of various pharmacological interventions, the wide heterogeneity in the presentation and pathophysiology of CS as well as the limited availability of high-quality evidence from randomized controlled trials lead to a lack of consensus regarding specific management protocols of these patients. Therefore, the use of inotropes and vasopressors varies widely, as the decision to adopt these drugs is based on local protocols derived from both a pathophysiological approach, as well as the limited evidence available. Mechanical support was needed by over one-third of the patients, with p-LVAD (Impella CP) being the most frequently used form of assistance. This finding aligns with evidence from various medical centers around the world and has been more commonly used since the introduction of p-LVAD.^3,8,10^ The use of a pulmonary artery catheter in our study was low (16%) in line with the data from the Altshock-2-Registry.^11^ The use of a pulmonary artery catheter in patients with CS is being revitalized by recent positive analyses of large registries in the United States (US) US supporting its use to define the hemodynamic profile and helping individualize the treatment strategies for patients with CS.^15^ In the United States, the approach is often guided by algorithms developed and driven by interventional cardiologists, which can influence the decision to use pulmonary artery catheters.

In our real-world study, and despite our best efforts, the mortality rate among these patients remains alarmingly high, underlying the urgent need to properly stratify patients with CS and to find new management approaches, targeting the underlying pathophysiology of the disease.^2–6^ The short-term prognosis of CS is directly related to the severity of the hemodynamic disorder. Recently, the SCAI SHOCK classification, developed by the SCAIs, provided a standardized framework for stratifying patients with CS based on their clinical presentation and hemodynamic status.^13^ The SCAI SHOCK classification provides robust hospital mortality risk stratification as higher SCAI SHOCK stages are associated with worse outcomes and higher mortality rates. The high mortality and complication rate observed in our study is similar to other high-volume centers and other international centers, considering that we excluded patients with SCAI SHOCK stage A and B who have a significantly better prognosis compared with those patients presenting with a stage C or worse.^16–18^

In our study, nonsurvivors are older and have a higher prevalence of COPD, a higher inflammatory burden, elevated baseline lactate levels with a further increasing trend during the hospitalization, elevated left ventricular filling pressure, and RV dysfunction. These findings align with the available evidence.^19–29^

Several factors were identified as predictors of in-hospital death for all causes, such as age, lactate levels at admission, lactate increase in the first 48 hours, CRP levels, and TAPSE. However, only CRP levels, lactate levels at admission, and changes in lactate levels were found to be independent predictors of in-hospital all-cause death, according to multivariate analysis. A systemic inflammatory response syndrome occurs frequently in patients with CS in the absence of sepsis. A recent retrospective study analyzed data from 8995 patients admitted to the Mayo Clinic CICU between 2007 and 2015 and found that one-third of the patients in the CICU met clinical criteria for Systemic Inflammatory Response Syndrome (SIRS) at admission. These patients have higher illness severity and worse outcomes across SCAI shock stages.^20^ However, in the context of CS, the clinical response of SIRS might be nonspecific and mainly related to the acute critical state. In this regard, the assessment of CRP levels (not included in the definition of SIRS) might be of additive value and may contribute to identifying those patients with an actual systemic inflammatory involvement who are at higher risk of worse clinical outcomes. Furthermore, the release of pro-inflammatory cytokines and chemokines in response to cardiac injury leads to endothelial dysfunction, microvascular thrombosis, and oxidative stress, which further exacerbate tissue injury and organ dysfunction, progressing the shock state and the multiorgan failure.^30^

In our study, a CRP cut-off of ≥25 mg/L at admission showed a sensitivity of 82% and specificity of 64% for predicting in-hospital mortality. Emerging evidence suggests that inflammation, as evidenced by increased levels of related biomarkers, is strongly associated with adverse CS-related outcomes.^19–21^ Our data align with a recent study, which included 240 patients, in which CRP levels during ICU treatment were associated with 30-day all-cause mortality.^28^ Furthermore, an increase of CRP levels by at least 200% from day 1 to day 3 during ICU treatment was found to be associated with an increased risk of 30-day all-cause mortality in patients with AMI complicated by CS.^28^ These data need to be expanded prospectively in a larger population to assess whether they may also have therapeutic implications, as patients with higher CRP levels may require more aggressive pharmacological or invasive management strategies. Moreover, CRP levels may be used as an easily available laboratory biomarker to monitor response to therapy and guide treatment decisions. Furthermore, the pathogenic role of inflammation in CS may provide a rationale for developing novel therapies targeting the inflammatory response in these patients.^31^ Our study also found a moderate correlation between CRP at admission and delta variations in lactate, suggesting that elevated CRP levels reflect a greater degree of inflammation, which can lead to impaired tissue perfusion and oxygen utilization. Effective clearance of lactate indicates improved tissue perfusion and resolution of hypoperfusion, resulting in a decrease in the systemic inflammatory response.^32,33^

Arterial lactate is the most widely used point-of-care parameter in CS and is a surrogate marker of hypoperfusion. Baseline arterial lactate is a widely used parameter to predict CS severity and mortality. However, the changes in lactate over time have recently been shown to predict survival and outperform the baseline lactate levels in predicting early mortality in patients with CS.^28^

Our study has several limitations. This is a single-center study with a small sample size that may not have sufficient power to detect potential statistical differences between the comparison groups. The small sample size may also induce an overfitting of the multivariable analysis. Furthermore, we meticulously reviewed each case to confirm the diagnosis of CS, but we cannot exclude an underdiagnosis. The predictive value of CRP measured in the early phase of CS may be affected by the timing of symptoms onset, the patient's underlying comorbidities, and the potential interference of medications administered in the early phase. The lack of complementary parameters, rather than echocardiographic indices, limited the evaluation of RV systolic function in our study. Finally, our population includes mainly White patients, and therefore our results cannot be generalized to other ethnic groups.

## CONCLUSIONS

Our study on patients hospitalized for isolated CS in a high-volume Italian hospital revealed several key findings. First, the most frequent presentation of CS was de novo CS, with AMI being the most common cause, followed by VHD and myocarditis. Second, all patients were given inotropes and vasopressors, with dobutamine and noradrenaline being the most used drugs. Third, mechanical support was needed by over one-third of the patients, with p-LVAD (Impella CP) being the most frequently used form of assistance. Fourth, we observed a high in-hospital mortality rate of approximately 50%, emphasizing the severity and poor prognosis associated with this condition. Fifth, nonsurviving patients exhibited distinct characteristics compared with survivors, and we identified several predictors of in-hospital all-cause death including elevated CRP levels at admission, higher baseline lactate levels, and increase in lactate levels during the hospitalization. In addition, we observed that a CRP cut-off of 25 mg/L showed reasonable sensitivity and specificity for predicting in-hospital mortality, further supporting its potential role as a prognostic marker in CS.

Understanding the regional heterogeneity in epidemiology, clinical course, outcomes, and predictors of mortality in CS is crucial for clinicians. It can guide healthcare providers in recognizing patients who are at high risk. Moreover, it can help in identifying novel therapeutic targets and optimizing treatment strategies by focusing on medical approaches tailored to individual patient characteristics and the underlying pathophysiology of the disease.
